# A Modified Physical Disability Screening Model after Treatment in the Intensive Care Unit: A Nationwide Derivation-Validation Study

**DOI:** 10.3390/jcm11123251

**Published:** 2022-06-07

**Authors:** Malihe Sadat Moayed, Amir Vahedian-Azimi, Keivan Gohari-Moghadam, Mohammad Asghari-Jafarabadi, Željko Reiner, Amirhossein Sahebkar

**Affiliations:** 1 Trauma Research Center, Nursing Faculty, Baqiyatallah University of Medical Sciences, Tehran 1435916471, Iran; msmoayed@gmail.com; 2 Medical ICU and Pulmonary Unit, Tehran University of Medical Sciences, Tehran 1419733141, Iran; kgohari@tums.ac.ir; 3 Department of Statistics and Epidemiology, Faculty of Health, Tabriz University of Medical Sciences, Tabriz 5165665811, Iran; m.asghari862@gmail.com; 4 Department of Internal Medicine, University Hospital Center Zagreb, School of Medicine, University of Zagreb, 10000 Zagreb, Croatia; 5 Biotechnology Research Center, Pharmaceutical Technology Institute, Mashhad University of Medical Sciences, Mashhad 9177948954, Iran; 6 Applied Biomedical Research Center, Mashhad University of Medical Sciences, Mashhad 9177948564, Iran; 7 Department of Medical Biotechnology and Nanotechnology, Faculty of Medicine, Mashhad University of Medical Sciences, Mashhad 9177948564, Iran; 8 Department of Biotechnology, School of Pharmacy, Mashhad University of Medical Sciences, Mashhad 9177948954, Iran

**Keywords:** derivation–validation groups, education level, intensive care unit, physical disability, inability

## Abstract

Background: Many of the survivors of critical illnesses in the intensive care unit (ICU) suffer from physical disability for months after the treatment in the ICU. Identifying patients who are susceptible to disability is essential. The purpose of the study was to modify a model for early in-ICU prediction of the patient’s risk for physical disability two months after the treatment in the ICU. Methods: A prospective multicenter derivation–validation study was conducted from 1 July 2015, to 31 August 2016. We modified a model consisting of three risk factors in the derivation group and tested the modified model in the validation group. They were asked for their physical abilities before being admitted, two months after discharge from the ICU by a binary ADL staircases questionnaire. The univariate and multivariate logistic regression was used to modify physical disability components in the derivation data set. Receiver operating characteristic curves were used to determine the sensitivity and specificity of the threshold values in the validation group. Results: Five-hundred nineteen survivors were enrolled in the derivation group, and 271 in the validation. In multivariable analysis, the odds ratio (OR) of physical disability significantly increased with educational level ≤ elementary school (OR: 36.96, 95%CI: 18.14–75.29), inability to sit without support (OR: 15.16, 95%CI: 7.98–28.80), and having a fracture (OR: 12.74, 95%CI: 4.47–36.30). The multivariable validation model indicated that education level, inability to sit without support, and having a fracture simultaneously had sensitivity 71.3%, specificity 88.2%, LR+ 6.0, LR− 0.33, PPV 90.9, and NPV 64.9 to predict physical disability. Applying the coefficients derived from the multivariable logistic regression fitted on the derivation dataset in the validation dataset and computing diagnostic index sensitivity 100%, specificity 60.5%, LR+ 2.5, LR− 0.003, PPV 80.8, and NPV 100. The modified model had an excellent prediction ability for physical disability (AUC ± SE = 0.881 ± 0.016). Conclusions: Low education level, inability to sit without support, and having a fracture in a modified model were associated with the development of physical disability after discharge from ICU. Therefore, these clinical variables should be considered when organizing follow-up care for ICU survivors.

## 1. Introduction

Increased intensive care unit (ICU) use and improvements in critical care medicine have resulted in an ever-expanding population of survivors with critical illness [1]. After hospital discharge, a significant proportion of ICU survivors report long-term cognitive, psychiatric, and/or physical disability problems, which are known together as post-ICU syndrome (PICS) [2,3].

The development of disability after critical illness is a complex process that may cause impaired daily functioning, 20–36% of survivors experienced job loss, 5–84% deteriorated employment status, and 17–66% occupation changes [4] that reduced their quality of life [5]. After a critical illness, the term (PICS) was proposed to recognize the presence of one or more deteriorations and impairments [6].

For improving long-term outcomes, such as ICU follow-up clinics and home-based rehabilitation programs, different ICU follow-up programs have been proposed for patients [7]. The extra effort, time, and care involved in developing a multidisciplinary management program can improve the long-term performance, capacity, and quality of life of ICU survivors as well as their families [8,9]. Moreover, it is known that the appropriate patient selection and the efficacy of interventions after ICU discharge could improve outcomes [2,10]. Patients who are at high risk for physical disability after treatment in the ICU, rather than all patients, will increase the chance of showing treatment effects in interventional studies [11]. Besides, follow-up of all patients would be less cost-beneficial than the follow-up of high-risk patients. The process of recovery may potentially be improved by early identification and rehabilitation in high-risk patients [12].

However, there is limited evidence on which clinical variables are associated with an increased risk of developing physical disability. Identifying these risk factors is important for the organization of targeted care such as ICU follow-up care and, eventually, for reducing healthcare expenditures. Schandl et al. developed a model that was based on four risk factors, which predict physical disability in ICU survivors. These factors were education level, < elementary school (ask patient or next-of-kin), reduced core stability (inability to sit without support in ICU), fractures, and the length of stay in ICU > 2 days [12]. Due to the importance of this issue and lack of a predictive model in Iranian patients, we assessed the potential predictors for subsequent physical disability in the Iranian population. Using a comprehensive and complete review of the literature on Iranian as well as international studies, we prepared and reviewed a list of risk factors for physical morbidity after critical illness. Finally, we came across three (education level ≤ elementary school, inability to sit without support in ICU, and having fracture) of the four risk factors that Schandl et al. [12] included in their model. The length of stay in the ICU for more than 2 days as a fourth risk factor was not applicable for our patient population because all patients who were admitted to the ICU were hospitalized for more than two days. Therefore, by eliminating this item, we tested the three-component model in the derivation group and confirmed the derivative results in the validation group. This study aimed to modify and validate such a model for early in-ICU prediction of the Iranian patient’s risk for physical disability two months after the treatment in the ICU.

## 2. Methods

### 2.1. Study Design and Setting

We performed a prospective derivation–validation cohort of patients discharged from 45 medical centers across 15 Iranian provinces from 1 July 2015, to 31 August 2016 (Supplementary File S1). All included patients in the study were registered in a list for two months follow-up post-ICU. All survivors of the intensive care unit of these 9 selected hospitals in each region were eligible to participate in the study if they met all inclusion criteria. Inclusion criteria included; (a) age ≥ 18, (b) discharge from ICU to the general ward, (c) absence of prior cognitive impairment, and (d) willingness to participate in this study. However, patients were excluded from the study (a) if they were briefly admitted for invasive procedures only (such as placement of epidural catheters or central venous lines), (b) if readmitted to the ICU, (c) if they died and (d) if they depended on ventilator.

In this study, 45 medical centers across 15 provinces of Iran were randomly selected through a multistage stratified sampling method and simple random sampling method. Patients were consecutively enrolled in the study and ICUs were selected by random sampling, taking into account type and location (Table 1). In each area, which included three provinces and nine hospitals, data were collected from eligible patients by local caregivers under the supervision of a lead clinical researcher. Eligible patients according to inclusion criteria were screened by principal clinical researcher and patients’ demographic characteristics (including age and gender) and ICU-related variables (including APACHEII, pre-ICU length of stay, ICU length of stay) of eligible patients were collected by trained local caregivers under the supervision of a lead clinical researcher.

The study was approved by the Institutional Review Board and the Ethics Committee of the Baqiyatallah University of Medical Sciences (Tehran, Iran) (code: IR.BMSU.REC.1395.217). Study participation was optional for participants. Informed consent was obtained from the patient, legal guardian, or healthcare surrogate, or a designated healthcare proxy.

### 2.2. Sample Size

The sample sizes were determined using data obtained from a pilot study (unpublished): derivation (*n* = 25), and validation (*n* = 45). Calculations were performed using G*Power version 3.0.10 (Universität Düsseldorf, Düsseldorf, Germany; available online at http://www.psycho.uni-duesseldorf.de/abteilungen/aap/gpower3/, last accessed on 26 February 2022) [13,14]. Calculations were performed for the variable ADL staircase, with an effect size of 0.4. With an alpha level of 5%, a confidence level of 95%, a power of 90%, and anticipated attrition of 10%, sample sizes of at least 483 and 251 patients were needed in the derivation and validation groups, respectively. However, we have chosen a sample size of 519 and 271 patients for derivation and validation groups because of possible drop-outs. We first selected the patients in the derivation groups, and when the patients in this group reached the desired number, we selected the patients in the validation group.

### 2.3. Data Collection

Demographic and clinical characteristics of patients including age (year), gender (male and female), severity of illness based on Acute Physiology and Chronic Health Evaluation (APACHE) II, Pre-ICU length of stay (days), ICU length of stay (days), Post-ICU length of stay and type of ICU were collected for each patient.

To assess the physical condition of patients, we used the ADL staircase questionnaire. The ADL-staircase consists of ten items and is an extended version of the Katz ADL index. Besides the Katz index’s six items (bathing, dressing, toileting, transferring, continence, and feeding) evaluating personal ADL, the ADL-staircase contains four items regarded as in-submental ADL (cooking, shopping, transportation, and cleaning). Each activity was evaluated concerning the patient’s ability to perform the activity independently or not. Patients were scored yes/no for independence in each of the ten functions. Total Katz ADL index score is in the range of 0 to 10 where score 10 represents an independent patient and 0 indicates a very dependent one [15].

Initially, the ADL staircase questionnaire was completed at the time of admission by a person very close to the patient (first-degree relatives or very close friends) who knew about the patient’s health and physical condition at the time before admission to the ICU. In addition, two months after ICU discharge, 519 and 271 ICU survivors received the questionnaire ADL staircase in the derivation and validation groups by email (20.6% vs. 22.5%), home visiting (56.8% vs. 54.2%), and message on smartphone (22.5% vs.23.2%). All patients were followed for completing the questionnaires by telephone one week after sending the questionnaire. The mean time for patients to complete the questionnaire and return it to the authors was 2.55 ± 0.96 months. Completed forms were returned to the authors in the study-provided sealed envelopes. A three-investigators panel evaluated de-identified questionnaires for inclusion based upon data completeness. Finally, the status of patients was compared with their status before ICU admission.

### 2.4. Statistical Analyses

Data were presented using mean (SD), median (min–max) for the Numeric Normal and non-normal variables, respectively, and frequency (percent) for categorical variables. Whole data was split into two subsamples: derivation and validation data. The between groups (derivation and validation) comparisons of numeric and categorical variables were made by independent t-test and Chi-square tests, respectively. The predicators education level ≤ elementary school, inability to sit without support and having fracture were included in the univariable and multivariable logistic regression of modified physical disability components in derivation data set. The status of patients was compared with their status before ICU admission based on ADL score two months after discharge from ICU. Patients were divided into two groups; the patients who got worse (the Katz ADL index score was lower than before admission to the ICU, which indicated that they were more dependent) and patients without changes (whose Katz ADL index score was unchanged from the one before ICU admission). The results showed that 347 (43.9%) patients had no changes and 443 (56.1%) patients were getting worse. Therefore, binary ADL staircases were used in both univariable and multivariable models. In both models, odds ratio (OR) and their 95% confidence interval (CI) were reported as the effect size of association. Multivariable binary logistic regression analysis was adjusted concerning age, gender, APACHE II score, pre-ICU length of stay, ICU length of stay and post-ICU length of stay.

Based on the coefficients derived from the model in the derivation dataset, the scores were computed for the validation data set. The model validation was assessed in the validation data set using diagnostic accuracy measures and their 95% CI, including sensitivity (SN), specificity (SP), positive predictive value (PPV), negative predictive value (NPV), positive likelihood ratio (LR+), negative likelihood ratio (LR−) and area under the curve (AUC). In the tables with zero counts, likelihood ratios were estimated using the substitution formula and the 0.5 was added to all cell frequencies before calculation. In all analyses, *p* values less than 0.05 were considered as significant. All analyses were conducted using IBM SPSS Statistics 20 (IBM SPSS Statistics, Armonk, NY, USA) and STATA software [ver.13] (StataCorp, College Station, TX, USA).

## 3. Results

**Figure 1:**
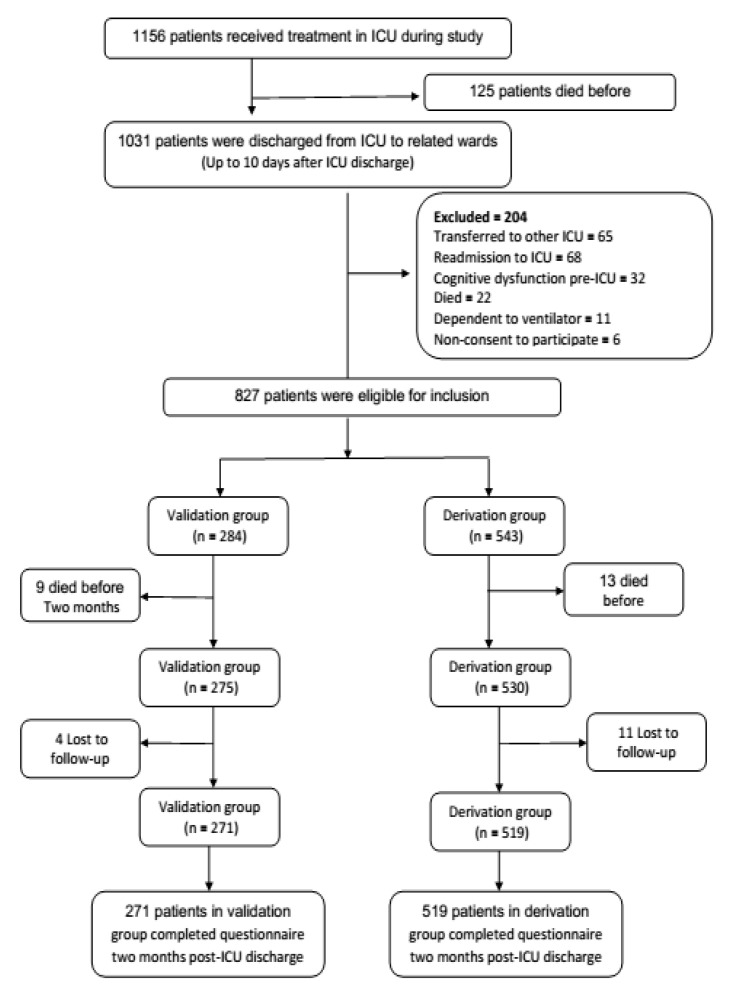
Flow diagram for patients who were included and excluded from the study. ICU: intensive care unit.

All patients discharged from the ICUs during a thirteen months’ period were consecutively enrolled in the study (Figure 1). According to flow chart for patients included and excluded from the study (Figure 1), 1156 patients were treated in 45 medical centers in 15 provinces of Iran during the study, of which 1156 patients were discharged from the ICU (125 patients died during this period). Overall, 204 patients out of 1031 patients who were discharged from the ICU were excluded from the study because they were not meeting the inclusion criteria—they were (a) transferred to other ICUs (*n* = 65), (b) readmitted to the ICU (*n* = 68), (c) had cognitive dysfunction before admission to the ICU (*n* = 32), (d) they died (*n* = 22), (e) they required mechanical ventilation to be continued after ICU discharge (*n* = 11), or (f) they did not consent to participate (*n* = 6). The remaining 827 eligible patients were divided into the derivation (*n* = 543) and validation (*n* = 284) groups. During the follow-up period, 13 patients (nine patients died, and four patients were lost the follow-up) and 24 patients (13 patients died, and 11 patients were lost the follow-up) were excluded from the validation and derivation groups, respectively. Finally, 519 survivors were enrolled in the derivation cohort, and 271 in the validation cohort. The distribution of demographic and clinical variables is presented in Table 2. Results indicated a consistency of the distribution of the variables between two groups. There were no significant differences between derivation and validation groups concerning the distribution of age, APACHEII score, pre-ICU length of stay, ICU length of stay, post-ICU length of stay, gender, questionnaire delivering, and ICU type.

The status of patients was compared with their status before ICU admission based on ADL score two months after discharge from ICU. Based on this comparison, patients were divided into two groups; the patients who got worse (the Katz ADL index score was lower than the one before admission to the ICU, which indicated that they were more dependent) and patients without changes (whose Katz ADL index score was unchanged from the one before ICU admission). The results showed that the 347 (43.9%) patients had no changes and 443 (56.1%) patients were getting worse. Table 3 shows the distribution of three predictor variables “education level”, “having a fracture”, and “inability to sit without support” according to binary ADL staircases in derivation group.

The results of univariable and multivariable logistic regression modified physical disability components in the derivation group are presented in Table 4. There were significant and positive associations between education level, inability to sit without support and having a fracture with binary ADL Staircases in both univariable and multivariable models, so that the odds of getting worse in ADL Staircases was (OR: 11.44, 95%CI: 6.97–18.78), (OR: 36.96, 95%CI: 18.14–75.29) and (OR: 4.79, 95%CI: 3.27–7.02), (OR: 15.16, 95%CI: 7.98–28.80), and (OR: 13.45, 95%CI: 5.55–35.05), (OR: 12.74, 95%CI: 4.47–36.30) for education level ≤ elementary school, inability to sit without support and having fracture in univariable and multivariable models, respectively.

### 3.1. Diagnostic Indices of the Multivariable Model of Modified Physical Disability Components in Derivation Sample

Diagnostic indicators of the multivariable validation model suggested that the multivariable model containing education level, inability to sit without support, and having fracture simultaneously had SN equal to 71.3, 95% CI (66.0–76.2), SP equal to 88.2, 95% CI (82.8–92.4), LR+ equal to 6.0, 95% CI (4.1–8.9), LR− equal to 0.33, 95% CI (0.27–0.38), PPV equal to 90.9, 95% CI (86.7–94.2), and NPV equal to 64.9, 95% CI (58.8–70.6).

### 3.2. Multivariable Logistic Regression Results of Modified Physical Disability Components in Validation Group

Applying the coefficients derived from the multivariable logistic regression fitted on the derivation group in the validation group and computing the diagnostic indices of the univariable validation model indicated that the multivariable model containing education level, inability to sit without support and having fracture simultaneously had SN equal to 100.0, 95% CI (98.9–100.0), SP equal to 60.5, 95% CI (53.3–67.4), LR+ equal to 2.5, 95% CI (2.1–3), LR− equal to 0.003, 95% CI (0.0001–0.410), PPV equal to 80.8, 95% CI (76.6–84.5), and NPV equal to 100.0, 95% CI (96.9–100) (Table 5).

## 4. Discussion

Survivors of critical illnesses often experience poor outcomes after hospitalization. This nationwide derivation–validation study assessed the relative contribution of risk factors concerning physical disability in a mixed ICU population and used this information to modify the model for early screening of physical disability in ICU survivors. A significant and positive association between low education level, inability to sit without support, and having a fracture with ADL was found, as well as getting the odds of getting worse ADL. In previous studies, lower educational level has been also associated with worse long-term survival after critical illness [16]. Needham et al. presented three major topics that must be considered to improve care and outcomes for intensive care survivors: (1) raising awareness and education, (2) understanding and addressing barriers to practice, and (3) identifying research gaps and resources [6]. Von Korff et al. demonstrated that patients with chronic spinal pain have a lower educational level [17], and educational level was a determinant of recovery after discharge [18]. Another predictor variable was the inability to sit without support and having a fracture associated with physical disability. If patients were unable to sit independently at the bedside of their ICU bed, we considered that the patient had poor stability. Ferrante et al. in a longitudinal study showed that pre-ICU disabilities were correlated with increased post-ICU physical disabilities in ICU survivors. It seems to be clear that former disabilities may provide prognostic information about post-critical illnesses outcomes [19]. Nordon-Craft et al. implemented physical therapy intervention including education, positioning, therapeutic exercise, and functional mobility training for ICU survivors. The interventions had significantly positive effect on patients’ ICU discharge outcomes [20]. Nevertheless, another study has demonstrated that physical intervention after discharge from ICU was not effective for improving physical disability outcomes [21].

Pain related fracture was reported in 62.7% of patients one year after major trauma, leading to instability as a risk factor for long-term physical disability [22]. Therefore, patients with facture need a longer time than non-trauma ICU patients to show similar degree of improvement [23].

Low educational level, inability to sit without support, and having a fracture together were three risk factors upon which our model was based. Fan et al. reported that ICU survivors who have evidence of muscle weakness get better within 12 months. They have confirmed that muscle weakness was associated with substantial impairment in physical function. They used several tests to assess muscle strength including handgrip, respiratory muscle strength, anthropometric measurement, and 6 min walk distance. Despite significant association between their variables with physical disability, they believed that these methods were not a reliable indicator for other neuromuscular factors, which may have an important impact on physical function, such as pain and endurance [24]. This study was done to investigate patients’ characteristics causing physical disability. Van Beusekom et al. demonstrated that several ICU related clinical variables such as length of stay in ICU, mechanical ventilation, acute physiology score, glucose score, and especially the reason for admission to ICU were associated with the development of disability conditions that should be taken into consideration as inclusion criteria for follow-up care [25]. Moreover, van Beusekom et al. demonstrated that ICU patients were five-fold more likely to develop a chronic condition during one year after ICU admission. Additional metabolic problems such as high plasma cholesterol, diabetes mellitus, and other cardiac, pulmonary, and neurologic diseases are the most prevalent newly developed chronic conditions in the ICU population during the year after admission in ICU [26]. Other studies could not show any significant correlation between increased blood glucose level and corticosteroid dose with muscle weakness; muscle strength was 3–11% lower for every additional day of bed rest. In addition, older age and the number of ICU days alert were significantly associated with peripheral muscle weakness [24].

Evidence-based data of the physical problems of PICS is required for improving the quality of rehabilitation services for patients who have PICS. This information is needed to inform healthcare providers, ICU survivors who have PICS and their family members to understand the kind of problems that they should anticipate [27].

The big advantage of this study is that it is unique since the data originated from different parts of Iran covering virtually the whole country. The study was performed in 45 medical centers across 15 Iranian provinces in different ICUs including adult medical, surgical, mixed, cardiovascular, burns, and toxicological with multistage cluster random sampling. The modified physical screening model was developed in the derivation population and validated in the validation population. For measuring activity daily living status before admission in the ICU and after discharge from ICU, a 10 item ADL-staircase was used.

The possible limitation of this study is that measuring physical disability before patients’ admission in ICU was retrospective. Therefore, some risks of recall bias might be possible since patients or next-of-kin were asked to estimate their Pre-ICU ADL status after ICU admission. Although cognitive dysfunction in ICU may have play a role in the trajectory of physical recovery and rehabilitation after ICU discharge, in the study the cognitive dysfunction was not monitored which is also a limitation of this study.

## 5. Conclusions

This prospective multicenter derivation-validation study demonstrated that three risk factors together in a developed model could predict physical disability in ICU patients after discharge. The results showed that low education level, inability to sit without support, and having a fracture were associated with the development of physical disability after discharge from ICU. Therefore, these clinical variables should be considered when organizing follow-up care for ICU survivors. Although this study reflects the situation in Iran, the same model might be applied in other countries, which have the same or similar type of healthcare organization.

## Figures and Tables

**Table 1:** Criteria for sampling hospitals for data collection.

Sampling Methods	Sampling Unit	Description of the Criteria
Categorization *	Regions	Thirty one provinces of Iran were divided into 5 regions; North, South, East, West, and Center of Iran
Stratified sampling	Provinces	Three provinces have been sampled for each region; Northern provinces (Golestan, Mazandaran, and Gillan); southern provinces (Fars, Hormozgan, Sistan and Balochestan); eastern provinces (North Khorasan, South Khorasan and Razavi Khorasan); western provinces (Kermanshah, East Azarbayejan, and Hamadan); and central provinces (Tehran, Isfahan, and Yazd)
Simple random sampling with replacement	Hospitals	Three hospitals in each province and 9 hospitals in each region were sampled
Simple random sampling	ICUs within a hospital	ICU was defined as adult mixed medical-surgical, medical, surgical, cardiovascular, burns, and toxicological
Simple random sampling	ICU beds within ICUs	Depending on number of beds in ICU, 5–8 subjects were enrolled in per ICU

* In this division, an attempt has been made to cover the whole country. ICU: intensive care unit.

**Table 2:** Demographic and clinical data.

Variables	Total (*n* = 790)	Derivation Group (*n* = 519)	Validation Group (*n* = 271)	*p*-Value
Age Mean ± SD	61.20 ± 11.14	61.47 ± 10.88	60.68 ± 11.63	0.343 *
APACHEII	21.39 ± 2.19	21.35 ± 2.11	21.48 ± 2.35	0.449 *
Pre ICU length of stay	4.45 ± 1.71	4.86 ± 1.63	4.66 ± 1.58	0.101 *
ICU length of stay	11.62 ± 3.73	11.76 ± 3.44	11.35 ± 4.17	0.175 *
Post ICU length of stay	6.63 ± 3.92	6.78 ± 3.53	6.34 ± 4.10	0.127 *
Gender, Female *n* (%)	444 (56.2)	287 (55.3)	157 (57.9)	0.497 **
Questionnaire delivering, by home visit *n* (%)	333 (42.2)	295 (56.8)	147 (54.2)	0.759 **
ICU Type				0.550 **
Burns, *n* (%)	45 (5.7)	30 (5.8)	15 (5.5)	
Trauma, *n* (%)	129 (16.3)	77 (14.8)	52 (19.2)	
General surgery, *n* (%)	186 (235)	126 (24.3)	60 (22.1)	
Open heart, *n* (%)	137 (17.3)	87 (16.8)	50 (18.5)	
Toxicological, *n* (%)	115 (14.6)	74 (14.3)	41 (15.1)	
Brain surgery, *n* (%)	110 (13.9)	75 (14.5)	35 (12.9)	
Medical, *n* (%)	68 (8.6)	50 (9.6)	18 (6.6)	

* Independent sample t-test. ** Chi-Square.

**Table 3:** Three predictor variables “education level”, “having a fracture”, and “inability to sit without support” according to binary ADL staircases in derivation group.

Variables		Total (*n* = 519)	Binary ADL Staircases		*p*-Value
			No Change (*n* = 195)	Getting Worse (*n* = 324)	
Having education	Yes (%)	214 (41.2)	22 (11.3)	192 (59.3)	<0.001
	No (%)	305 (58.8)	173 (88.7)	132 (40.7)	
Having a fracture	Yes (%)	92 (17.7)	5 (2.6)	87 (26.9)	<0.001
	No (%)	427 (82.3)	190 (97.4)	237 (73.1)	
Inability to sit without support	Yes (%)	291 (56.1)	64 (32.8)	227 (70.1)	<0.001
	No (%)	228 (43.9)	131 (67.2)	97 (29.9)	

**Table 4:** The univariable and multivariable logistic regression results of modified physical disability components in derivation group.

Predictors	Univariable	Multivariable	AUC ± SE
	OR (95% CI)	B, OR (95% CI)	
Education level ≤ elementary school	11.44 (6.97–18.78)	36.96 (18.14–75.29)	0.89 ± 0.014
Inability to sit without support	4.79 (3.27–7.02)	15.16 (7.98–28.80)	
Having fracture	13.45 (5.55–35.05)	12.74 (4.47–36.30)	

B: Regression coefficient; OR: Odds Ratio; AUC: area under the curve; SE: standard error; AUC based on multivariable logistic regression model. Binary ADL Staircases as dependent variable was used in both univariable and multivariable models, Goodness of fit, Hosmer–Lemeshow test, chi^2^ (130.89), *p*-value < 0.05, Model sensitivity: 100%, Specificity: 60.5%, Accuracy: 85.2%.

**Table 5:** Multivariable logistic regression results of modified physical disability components in validation group.

Education level ≤ elementary school	AUC ± SE 0.881 ± 0.016
Inability to sit without support	
Having fractures	

## Data Availability

Data are available from the first and corresponding authors upon a request.

## Supplementary Materials

The following supporting information can be downloaded at: https://www.mdpi.com/article/10.3390/jcm11123251/s1, File S1: Three predictor variables “education level”, “having a fracture”, and “inability to sit without support” according to binary ADL staircases in in derivation group. File S2: TRIPOD Checklist: Prediction Model Development and Validation.
