# Supplementary material for: A Modified Physical Disability Screening Model after Treatment in the Intensive Care Unit: A Nationwide Derivation-Validation Study
Source: J Clin Med. 2022 Jun 7;11(12):3251. doi: 10.3390/jcm11123251 (PMC9224861; doi:10.3390/jcm11123251)
Supplement: Supplementary file 1 [file jcm-11-03251-s001.zip › Supplementary 1.pdf]

TS1: Three predictor variables “education level”, “having a fracture”, and “inability to sit without support” according to binary ADL staircases in in derivation group

Variables		Total (n=519)	Binary ADL staircases		*P*-value
			No change (n=195)	Getting worse (n=324)	
Having Education	Yes (%)	214 (41.2)	22 (11.3)	192 (59.3)	<0.001
	No (%)	305 (58.8)	173 (88.7)	132 (40.7)	
Having a fracture	Yes (%)	92 (17.7)	5 (2.6)	87 (26.9)	<0.001
	No (%)	427 (82.3)	190 (97.4)	237 (73.1)	
Inability to sit without support	Yes (%)	291 (56.1)	64 (32.8)	227 (70.1)	<0.001
	No (%)	228 (43.9)	131 (67.2)	97 (29.9)	
